# Advanced Ovarian Cancer during Pregnancy. Tumour Evolution Analysis and Treatment Approach

**DOI:** 10.3390/medicina57050426

**Published:** 2021-04-28

**Authors:** Ana Isabel Bueno Moral, Jose Carlos Vilches Jiménez, Adriana Serrano Olave, María Pilar Espejo Reina, María Estrella Valdivia de Dios, Jesús S. Jiménez López

**Affiliations:** Department of Obstetrics and Gynaecology, Regional University Hospital, 29011 Málaga, Spain; jovilo90@gmail.com (J.C.V.J.); adrianaserranoolave@hotmail.es (A.S.O.); pilarespejoreina@gmail.com (M.P.E.R.); doctoravaldivia@yahoo.com (M.E.V.d.D.); jesuss.jimenez.sspa@juntadeandalucia.es (J.S.J.L.)

**Keywords:** pregnancy, ovarian cancer, chemoradiation, cancer, surgery

## Abstract

Background: The possible presence of malignant adnexal mass should be considered during pregnancy. For this reason, it is important to keep in mind such possibility while performing routine obstetric ultrasounds to diagnose asymptomatic ovarian cancer in the early stages. Case presentation: 27-year-old pregnant patient with a known adnexal tumour occurring at week 20 and enlarged supraclavicular lymph nodes of 3 cm size who was diagnosed with metastases from low-grade papillary serous ovarian carcinoma. The patient, obstetricians, neonatologists and oncologists agreed on initiating neoadjuvant chemotherapy and performing an elective C-section at week 34. She gave birth to a female infant weighing 2040 g who is currently in good health, and continues receiving follow-up care by a medical oncologist. Conclusions. An early diagnosis of gynaecologic malignancies during pregnancy is of critical importance because, although they are very rare, managing and treating carcinomas at an early stage allow us to increase maternal and fetal well-being and to offer more alternatives to our patients.

## 1. Introduction

In clinical practice, adnexal tumours are incidental findings during pregnancy which have been noted to occur in 2.3% and 4.1% of all cases [1]. Most of these tumours are benign and associated with pregnancy. They also have spontaneous resolution. 10% are benign tumours such as cystadenomas, dermoid cysts and endometriomata [1,2]. These lesions can result in torsion, rupture and bleeding, as well as present a risk of malignancy, which should be noted [1,3].

Within adnexal tumours, the incidence rate of malignancies is 6% and, for this reason, a deeper exploration of adnexal masses is needed to avoid any diagnostic delays, particularly of those persistent [1,4].

Most of these tumours are clinically asymptomatic (65–80%). When patients present symptoms, these correspond with those of pregnancy [1,5]. Thanks to obstetric screening scans, ovarian cancer during pregnancy can be diagnosed in the early stages [1,5].

The association between cancer and pregnancy is very rare, with an incidence rate between 0.02% and 0.1% [1]. In developed countries, the maternal age has been increasing during the last years and, since cancer development is directly correlated with age, this is expected to result in an increased incidence rate in the future [1,6]. More frequent diagnoses are breast cancer and haematological cancer [1]. Within gynaecological malignancies, ovarian cancer ranks third after breast cancer and cervical cancer, with an incidence rate of 0.2–3.8% per 100,000 pregnancies [7].

Most of ovarian malignancies during pregnancy are linked to germ-cell tumours (40%), particularly dysgerminomas, and to epithelial tumours [6,8,9].

Currently no effective screening strategy is available for ovarian cancer. It is often diagnosed in advanced stages (67% in Stage III and IV). However, quite the opposite is the case during pregnancy, where diagnosis at Stage I (63%) seems to happen more often followed by diagnosis at Stage III (24%) [8]. The 5-year survival rate shows a direct correlation with the stage at diagnosis, which is 44% on average. Nonetheless, this rate hits 99% for patients diagnosed at Stage I [10].

For ovarian cancers diagnosed during pregnancy, all implications, complications and prognosis should be considered for both fetus and mother [1]. Any delays in surgical or medical treatment initiation must therefore be considered carefully since it could be harmful for the mother. Likewise, any preterm termination of pregnancy jeopardises fetal well-being.

## 2. Case Presentation

A 27-year-old, asymptomatic primigravida without any relevant personal or family medical history. She was receiving follow-up care during a year for bilateral adnexal masses of 3–4 cm size which were found during a routine gynaecological check-up and that may be indicative of bilateral teratoma. During the follow-up care period, we requested a tumour marker test and it was reported as normal. No changes were observed either.

**Figure 1:**
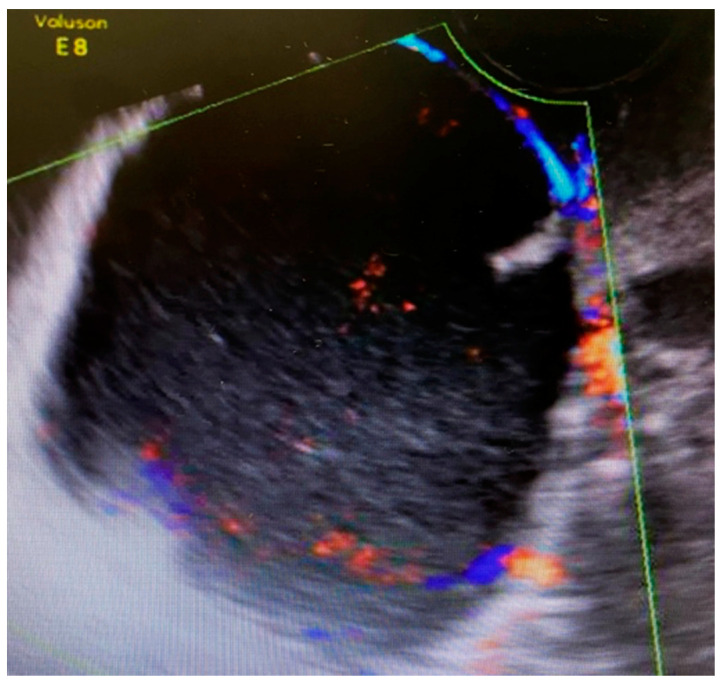
Ultrasound scan compatible with complex cystic formation.

The first 12-week ultrasound showed 66 mm × 48 mm cysts between the bladder and the cervix with peripheral vascularization, and another left paracervical cystic and heterogeneous lesion of 39 mm × 26 mm size (Figure 1). A follow-up visit was indicated requiring close monitoring.

No relevant morphologic findings were detected in the 20-week fetal ultrasound but the same described pelvic formations. Tumour markers were slightly elevated except CA (cancer antigen) 19.1, which was normal; CA 125: 38.3, CA 19.9: 18.6, HE-4 (Human Epididymal Protein): 79.7 and 20.56 for ROMA index (risk of ovarian malignicy algorithm index).

**Figure 2:**
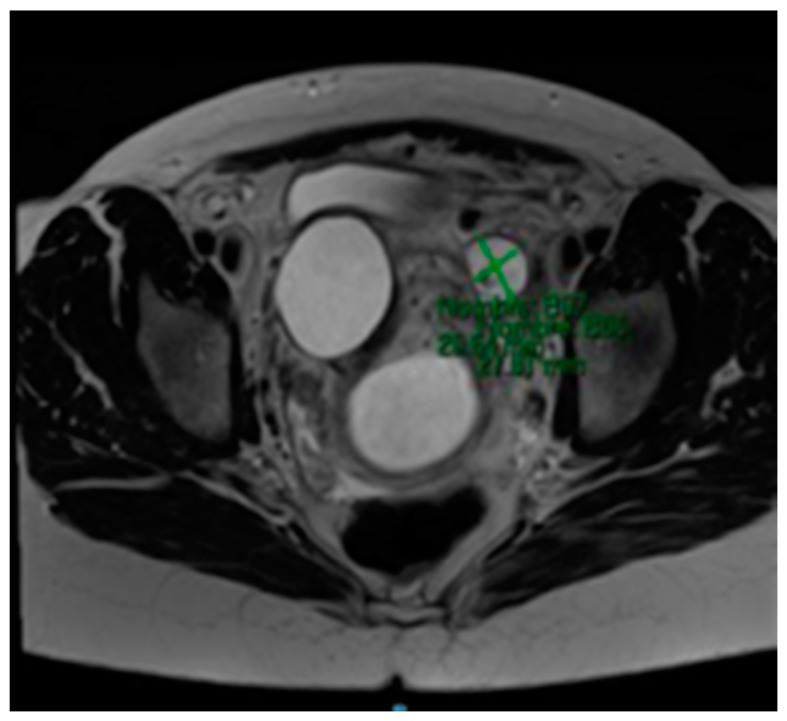
MRI scan compatible with 27 mm × 20 mm cystic formation in the left hemipelvis.

**Figure 3:**
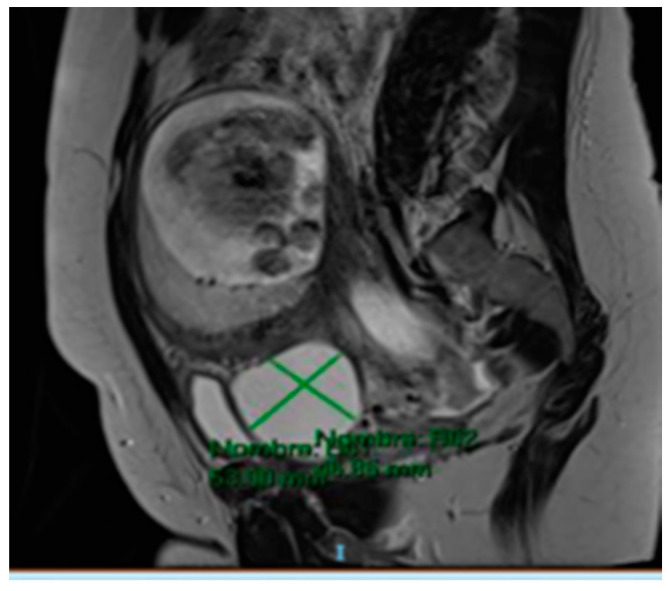
MRI scan compatible with 53 mm × 46 mm cystic formation in the right paracervical area.

The MRI (Magnetic Resonance Imaging) scan showed 53 mm × 46 mm thin-walled cysts with a septum in the right paracervical area, another 27 mm × 20 mm cystic formation in the left hemipelvis (Figure 2 and Figure 3) and presacral 2 cm cysts containing a solid pole.

At week 20, specialty consultation was indicated after self-appreciation of enlarged left supraclavicular lymph nodes measuring 3 cm. Therefore, a biopsy was performed showing metastases from low-grade papillary serous ovarian carcinoma.

Patient was referred for medical oncology evaluation and a multidisciplinary committee with neonatologists, oncologists, gynaecologists-oncologists and obstetricians was held to decide the best treatment option. We took into account patient’s preferences and it was agreed to initiate neoadjuvant chemotherapy with paclitaxel 175 mg/m^2^ every 3 weeks at week 28 and elective caesarean section at week 34 after two cycles.

She required hospitalisation at week 32 due to threatened preterm delivery, as well as tocolysis and fetal lung maturation.

Finally, at week 34, she was hospitalised for elective C-section and staging surgery. She gave birth a female infant weighing 2040 g with APGAR scores 5/8/10, who is currently in good health, and underwent classic cesarean section via laparotomy under general anaesthesia.

**Figure 4:**
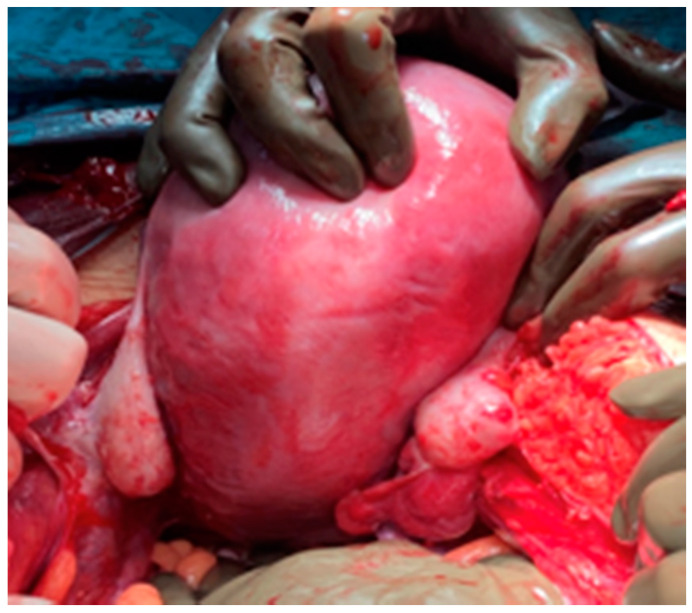
Macroscopic image of uterus and adnexa.

**Figure 5:**
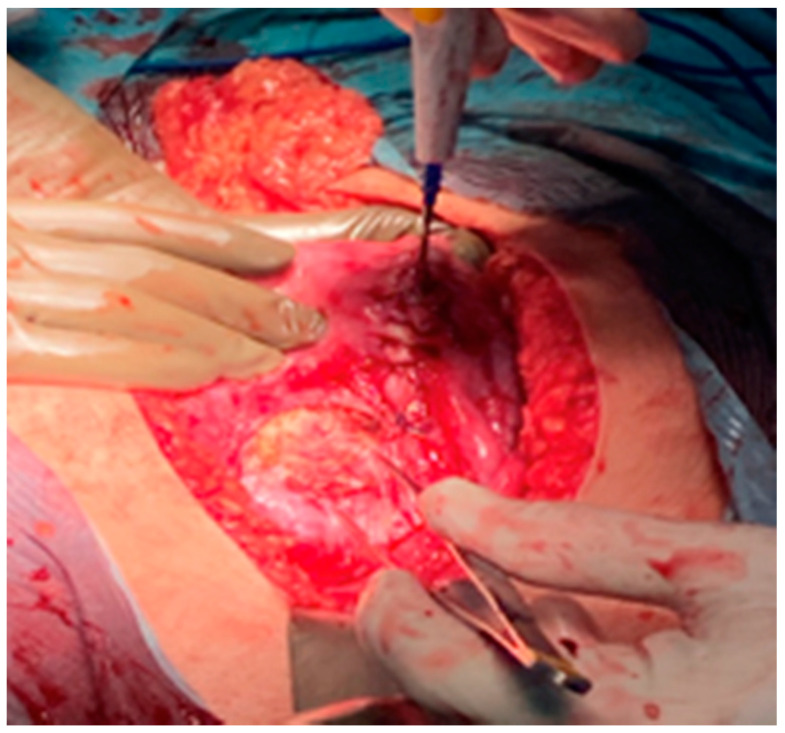
Macroscopic image of 2 cm implant in the anterior uterine wall.

During surgery, both ovaries and fallopian tubes appeared macroscopically healthy (Figure 4). An implant of 2 cm size was found in the anterior uterine wall (Figure 5) and another implant of smaller size was found in the pouch of Douglas. It was also observed a 2 cm size cyst located between the anterior uterine wall and vesical fold. Rest of the abdominal cavity was normal. Concordant results were obtained at intraoperative biopsy, so complete surgical excision was carried out to remove the abovementioned cyst. Total extrafascial hysterectomy, double adnexectomy and omentectomy were also performed as per the standard protocol without any incidents. Postoperative course was favourable and patient was discharged from hospital 5 days later.

Pathology test results revealed a low-grade papillary serous ovarian carcinoma that was located in the ovaries and infiltrated fallopian tubes and uterus (serous membrane, in the whole myometrial thickness and cervix) with extensive vascular invasion and omental infiltration, and the presence of 70% of estrogen receptors and 90% of progesterone receptors with a ki 67 antigen percentage score of 3–5%.

The patient is currently receiving follow-up care and has received 7 cycles of paclitaxel (175 mg/m^2^ every 3 weeks) and carboplatin (AUC 6-under the concentration-time). She also received negative genetic test results. Last evaluation with PET-CT (positron emission tomography-computed tomography).: enlarged left supraclavicular lymph nodes suggestive of infiltrations, and enlarged retrocrural, para-aortic, bilateral and right iliac fossa lymph nodes where malignancy can’t be ruled out.

The patient still had disease persistence at supraclavicular level after 15 radiotherapy fractions of 45 grays so she is receiving maintenance hormonal therapy with letrozole 2.5 mg daily. Currently, after 1 year of follow-up, patient has stable disease and no disease has been identified at the abdominal level or in other locations different from supraclavicular lymph nodes. The baby is now feeling well and hasn’t referred any relevant complication.

## 3. Discussion

Most of ovarian malignancies during pregnancy are diagnosed in the early stages, with disease still confined to the ovary, therefore allowing to perform conservative surgery.

However, we present the case of a patient who was diagnosed with low-grade ovarian tumour at Stage IV due to the appearance of supraclavicular metastases. Given this singularity, it was agreed to undergo neoadjuvant chemotherapy and surgery as per the standard protocol once fetal lung maturity is attained as described in studied literature [7], considering that the disease seemed to be confined to the ovary at the pelvic level and the possibility of performing cytoreduction surgery.

An ovarian cancer diagnosis during pregnancy represents a challenge for gynaecologists and obstetricians because it concerns both the mother and the fetus. The diagnosis is built on ultrasound findings reported in diagnostic imaging techniques such as MRI, where tumour markers values are limited for ovarian malignancies during pregnancy [1,2,4,11].

Low-grade papillary serous ovarian carcinoma is an infrequent malignancy representing just 5–8% of all ovarian cancers and it is usually diagnosed in patients with an average age of 45 years. This clinical entity requires a pathology-confirmed diagnosis due to the difficulty of making an accurate diagnosis using imaging techniques and distinguishing it from benign masses. Moreover, these tumours are usually detected in earlier stages and are confined to the ovary. This highlights the singularity of our case because it is a young patient suffering from low-grade papillary serous ovarian carcinoma with disseminated disease and lymph node involvement at diagnosis.

The complications include uterine rupture, hemoperitoneum, obstructed labour, torsion, recurrence, hypertension, rapid tumour progression, preterm delivery and fetal or maternal death.

It is important to inform the patient about the situation and potential treatment options. For more advanced stages, pregnancy termination before week 24 might be advised, and for patients who are more than 24 weeks pregnant, different approaches can be followed: surgery at 32 weeks of gestation or neoadjuvant chemotherapy in combination with surgery during C-section or after a term delivery [1,3,5]. However, it is critical to respect the patient’s decision once they have been informed. For this reason, there are certain cases where pregnancy is not terminated, such as our case report, despite diagnosis at such an advanced stage. The patient preferred to continue with gestation but showed a high level of anxiety as she reached a higher gestational age. For this reason, in addition to providing relevant psychological and emotional support, we agreed with the patient and the neonatal professionals to terminate pregnancy at week 34 after lung maturation and reaching optimal fetal viability after two cycles of neoadjuvant chemotherapy so that cytoreductive surgery could then be performed.

Nowadays, the two main pillars of standard treatment for ovarian cancer are surgery and chemotherapy. Chemotherapy should be administered after the 16th to 18th week [1,2,4,5]. On the basis of the available data, the use of chemotherapy is considered to be safe during pregnancy, although more research is needed in this regard [1,4,11].

Surgery allows to perform diagnosis, staging and cytoreduction and is indicated after the second trimester [2,5,6,12,13]. Given the high level of difficulty attached to this procedure, it should be performed by experts in surgery or gynaecological oncology [1,5]. In this case, patients are treated with a laparotomy approach although laparoscopy using minimal manipulation is indicated for certain patients with initial tumours [2]. In many instances, we rely on intraoperative studies to define an approach. No poor prognosis factors have been observed in patients undergoing surgery during pregnancy after the second trimester [2,5,6,9].

Following the same approach that we adopted for this patient, delaying intraparturm or postpartum surgery with chemotherapy neoadjuvant therapy can be an option in these cases [6]. Patients with epithelial ovarian cancer should receive the standard treatment with carboplatin and paclitaxel every three weeks although substantial placental transfer has been described with platinum compounds [6]. Bevacizumab is not indicated for this disease because of the potential teratogenic effects.

## 4. Conclusions

Diagnosis, treatment and monitoring of patients with ovarian cancer during pregnancy requires a multidisciplinary approach, taking into account the stage of disease, gestational age and patient desire in order to provide the best alternatives [5].

These diagnoses are of infrequent occurrence, but they should be suspected to be able to initiate an effective treatment. In addition to this, it is important to consider atypical presentations of ovarian cancer, as in our case report, to address this kind of situations at as early a stage as possible.

## Data Availability

All data generated or analysed during this study are included in this published article.
